# Lens Cytoskeleton: An Update on the Etiopathogenesis of Human Cataracts

**DOI:** 10.7759/cureus.56793

**Published:** 2024-03-23

**Authors:** Christina Karakosta, Martina Samiotaki, George Panayotou, Dimitrios S Papaconstantinou, Marilita M Moschos

**Affiliations:** 1 Ophthalmology, National and Kapodistrian University of Athens School of Medicine, Athens, GRC; 2 Proteomics, Biomedical Sciences Research Center "Alexander Fleming", Athens, GRC; 3 Ophthalmology, Gennimatas Hospital, National and Kapodistrian University of Athens School of Medicine, Athens, GRC; 4 1st Department of Ophthalmology, National and Kapodistrian University of Athens, Athens, GRC

**Keywords:** cytoskeleton, crystallin, aging, lens, cataract

## Abstract

A cataract is a loss of the transparency of a normal crystalline lens. Multiple factors, including age as the major risk factor for cataracts, can disturb the transparency of the crystalline lens due to cumulative damage from environmental insults to proteins, particularly crystallins. Lens proteins do not turnover, and crystallins undergo extensive post-translational modifications (PTMs) with age in order to interact with each other and maintain their soluble basis for lens transparency. These PTMs include truncation, oxidation, deamidation, acetylation, phosphorylation, and glycosylation. Cataract formation, apart from protein PTMs, involves protein crosslinking, protein insolubilization, and aggregation. Oxidation is a key feature in age-related cataract formation. Due to the role of genetic and environmental factors, as well as its variable clinical presentation, we consider cataracts to be a multifactorial disease. The preliminary results of our study indicate that proteins implicated in the pathway of a structural constituent of the eye lens (BFSP1, BFSP2, CRYAA, CRYAB, CRYBA, CRYBB, CRYGC, CRYGD, CRYGS, KRTs, and VIM), together with AQP1 and AQP5, may also be involved in lens aging.

## Introduction

A cataract is the loss of normal crystalline lens transparency. Cataract is responsible for 33% of visual impairment and 51% of blindness worldwide [1]. In 2005, 24.4 million people suffered from cataracts in the United States, and this number is expected to double by 2025 [1].

Based on the location of the opacity and LOCS III (Lens Opacities Classification System III), cataracts could be classified as nuclear sclerotic, cortical, posterior subcapsular, and anterior subcapsular (rare) [2]. Based on the origin, cataracts could be described as age-related (congenital, juvenile, pre-senile, senile), traumatic (including ocular trauma and previous intraocular surgery), drug-induced (i.e., corticosteroids, phenothiazine, amiodarone), or metabolic (diabetes mellitus) [1]. Risk factors for cataract formation include age, smoking, diabetes mellitus, exposure to ultraviolet light, hypertension, prolonged corticosteroid use, ocular trauma (including prior ocular surgery), genetic predisposition, high myopia, exogenous estrogen use, increased body mass index, and alcohol consumption [1].

The human crystalline lens consists of three parts: the capsule, the lens epithelium, and the lens fibers. The lens capsule is a basement membrane that entirely surrounds the lens. The lens epithelium is simply cuboidal and is present only at the anterior surface of the lens, between the capsule and the lens fibers [3]. The mitotic lens epithelial cells migrate toward the lens equator [3]. There, the elongated cells lose their nucleus and the majority of their organelles and differentiate into lens fibers [3]. Lens fibers migrate posteriorly and centrally as new cells are added superficially [3]. As a result, the center of the lens houses the older lens fibers. Lens fibers are densely packed and have little intercellular space [3].

The human lens has the highest protein concentration in the human body, approximately 33%.

Lens proteins are divided into water-soluble and water-insoluble groups [4]. The water-soluble proteins are mainly the crystallins, α-, β-, and γ-. However, β- and γ-crystallins are grouped together as a βγ-crystallin superfamily due to their similar structure [4]. Α-crystallins have two subunits, αA and αB, and belong to a small heat-shock protein family called chaperones, which associate with partially denatured proteins in order to prevent their aggregation [4]. The main role of α-crystallins seems to be the inhibition of the total denaturation and insolubilization of βγ-crystallins [4,5].

The water-insoluble proteins can be further divided into urea-soluble and urea-insoluble groups. The urea-soluble group consists of cytoskeletal proteins, and the urea-insoluble group consists of lens fiber cell membrane proteins. The urea-soluble (cytoskeletal) protein group includes the common microfilaments and microtubules, as well as the filament protein vimentin and the beaded filaments of the lens-specific proteins BFSP1 (filensin) and BFSP2 (CY49 or phakinin) [4]. The urea-insoluble proteins include a member of the aquaporin group, the major intrinsic protein (MIP or aquaporin 0), which is responsible for water transport, intercellular communication, and movement of ions in the lens [3,4].

The aim of this study is to present the preliminary data of our proteomic study and to discuss the pathogenesis of age-related cataracts, including the role of the main lens cytoskeletal proteins.

## Materials and methods

This study is part of a project. This study was conducted according to the ethical principles of medical research involving human subjects, according to the Declaration of Helsinki. Informed consents were obtained from all patients. The clinical data were assessed and anonymized for patients’ confidentiality. Ethical approval (18534/20-07-22) was granted by the Institutional Ethics Board of the Hospital. All patients presented with cataracts at the Ophthalmology Department were eligible for the study. Patients were divided into three groups. The inclusion criteria for group 1 (diabetic cataract, DC) were patients with DM (type 1 or 2), cataracts present during slit lamp examination, visual acuity lower than 20/40, and patients aged younger than 65 years. The inclusion criteria for group 2 (age-related cataracts, ARC) were patients without a history of DM, cataracts present during slit lamp examination, visual acuity lower than 20/40, and patients older than 75 years. Inclusion criteria for group 3 (post-vitrectomy cataract, PVC) were patients of any age without a history of DM, cataract present during slit lamp examination, vitrectomy surgery for retinal detachment in the last 12 months, and visual acuity lower than 20/40.

The exclusion criteria for all groups were a history of ophthalmic trauma, with or without an intraocular foreign body, and chronic use of cortisone, either topical or systemic. Table 1 shows the main demographic characteristics of the patients included in the study.

**Table 1:** The demographic data of the patients included in the study. SD: standard deviation; DC: diabetic cataract; ARC: age-related cataract; PVC: post-vitrectomy cataract.

Main characteristics	Group 1 (DC)	Group 2 (ARC)	Group 3 (PVC)
Number of subjects	11	12	7
Mean age (years, mean ± SD)	61.7±4.3	79.6±4.2	60±10.2
Sex (male/female)	7:4	5:7	2:5
Ethnicity (Caucasian/African)	8:3	12:0	7:0
Diet supplementary intake (yes/no)	3:8	5:7	1:6

Two sample types were collected from each patient: the anterior capsule and the content of the phacoemulsification (phaco) cassette. During cataract surgery, when capsulorhexis was completed, the anterior capsule of 5-5.5 mm diameter was collected using Utrata forceps through the main incision and stored in a sterile plastic box containing 1-2 ml of balanced salt solution (BSS). At the end of the surgery, the content of the phaco cassette was collected from the phaco machine (Centurion® Vision System, Camberley, UK). The phaco cassette contained BSS with the phacoemulsified pieces of the crystalline lens, along with the re-secreted aqueous humor. Both samples were stored in the freezer at −80 °C (Haier Biomedical, Qingdao, P.R. China) in the Microbiology Department of the Hospital. The samples were transferred to the lab using dry ice.

The protein extracts from both sample types were processed by tryptic digestion using the Sp3 protocol [6]. Samples were analyzed on a liquid chromatography-tandem mass spectrometry (LC-MS/MS) setup consisting of a Dionex Ultimate 3000 nanoRSLC coupled online with a Thermo Q Exactive HF-X Orbitrap mass spectrometer (Thermo Fisher Scientific, Inc., Waltham, MA).

Orbitrap raw data were analyzed in DIA-NN 1.8 (Data-Independent Acquisition by Neural Networks) by searching against the Human_Reviewed Proteome (downloaded from UniProt, 50,516 protein entries, April 11, 2022) using the software's library-free mode. The software allows up to two tryptic missed cleavages and a maximum of three variable modifications/peptides.

Software Perseus (version 1.6.15.0, Max-Planck Institute of Biochemistry, Bavaria, Germany) was used for statistics (defined groups, t-test) and data visualization. The raw intensities were Log2 transformed, classified into described clinical groups, filtered based on valid values set to 50%, and missing values were imputed based on normal distribution. 

Two sample t-tests were performed for the ARC-DC and ARC-PVC comparisons. Volcano plots were used to visualize the results of t-tests, with permutation-based FDR set at 0.05 and S0 (artificial within-group variance) set at 0.1. Additionally, an ANOVA test was performed for group comparisons. Heatmaps were used to visualize the results of the ANOVA test, with permutation-based FDR set at 0.05 and S0 (artificial within-group variance) set at 0.1.

## Results

The study included a total of 30 patients. Group 1 (DC) included 11 patients, group 2 (ARC) included 12 patients, and group 3 (PVC) included 7 patients.

In phaco cassette content samples, BFSP1 and specific crystallins (CRYBA1, CRYBA2, CRYBA4, CRYBB1, CRYBB2, CRYGC, CRYGD, and CRYGS) were upregulated in the DC and PVC groups compared to the ARC group. In the anterior capsule and phaco cassette content samples, the pathways downregulated in ARC groups included MIP, structural constituents of the eye lens (including CRYAA and CRYAB), microfilament motor activity, microtubule binding, and organizing center. In particular, the proteins implicated in the pathway of a structural constituent of the eye lens included BFSP1, BFSP2, CRYAA, CRYAB, CRYBA1/2/4, CRYBB1/2/3, CRYGC, CRYGD, CRYGS, KRTs, and VIM.

**Figure 1:**
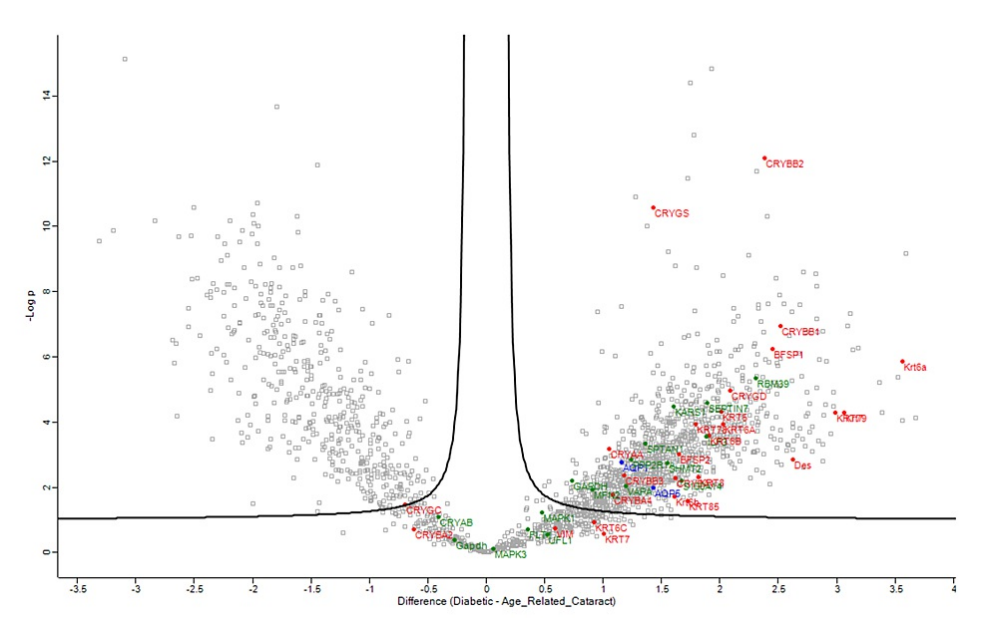
Volcano plot showing structural constituent of eye lens pathway (RED), microtubule cytoskeleton pathway (BLUE) and major intrinsic protein (MIP) pathway (GREEN) of anterior capsule samples in age-related cataract and diabetic cataract groups.

**Figure 2:**
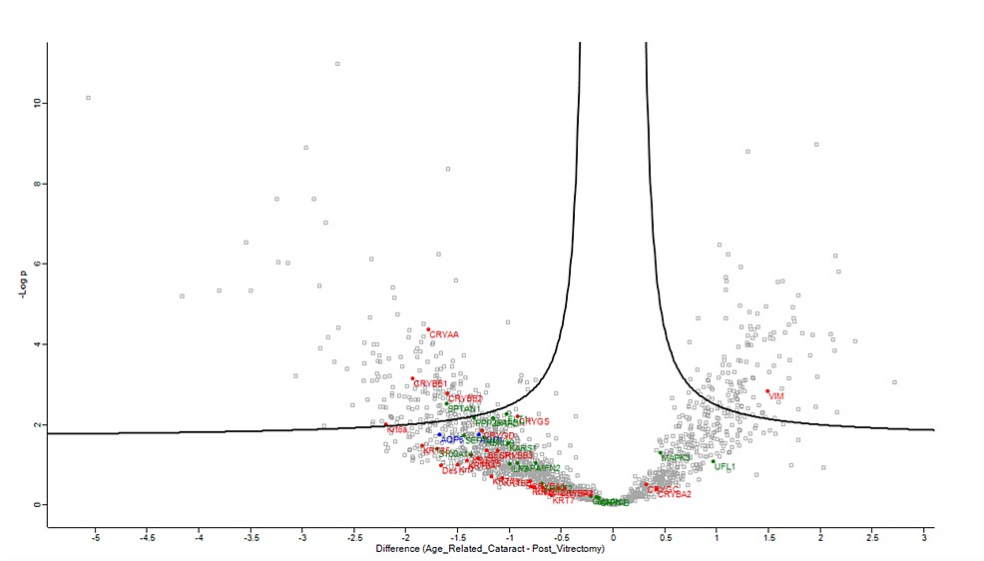
Volcano plot showing structural constituent of eye lens pathway (RED), microtubule cytoskeleton pathway (BLUE) and major intrinsic protein (MIP) pathway (GREEN) of anterior capsule samples in age-related cataract and post-vitrectomy cataract groups.

**Figure 3:**
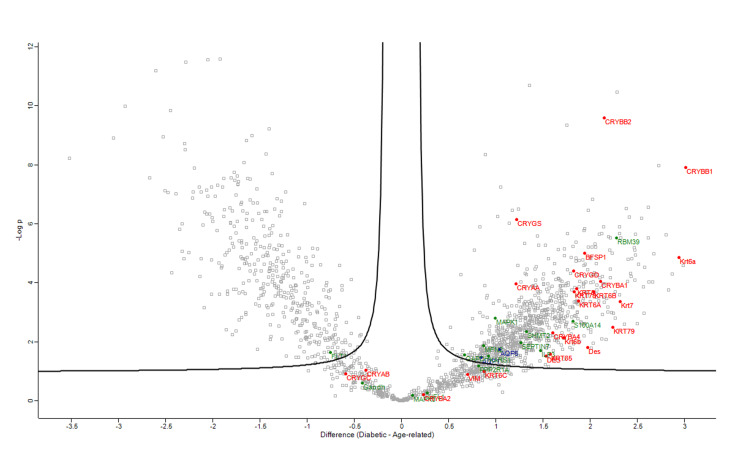
Volcano plot showing structural constituent of eye lens pathway (RED), microtubule cytoskeleton pathway (BLUE) and major intrinsic protein (MIP) pathway (GREEN) of phaco cassette content samples in age-related cataract and diabetic cataract groups.

**Figure 4:**
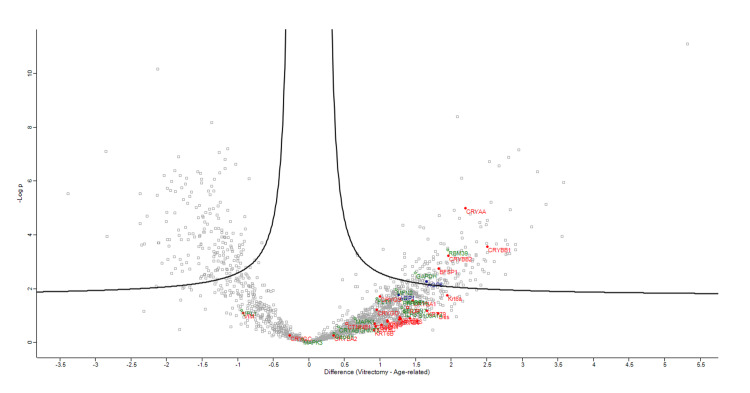
Volcano plot showing structural constituent of eye lens pathway (RED), microtubule cytoskeleton pathway (BLUE) and major intrinsic protein (MIP) pathway (GREEN) of phaco cassette content samples in age-related cataract and post-vitrectomy cataract groups.

Figures 1-4 demonstrate volcano plots of structural constituents of the eye lens pathway, microtubule cytoskeleton pathway, and MIP pathway of anterior capsule samples (top left and top right) and phaco cassette content samples (bottom left and bottom right) in the ARC, DC, and PVC groups.

**Figure 5:**
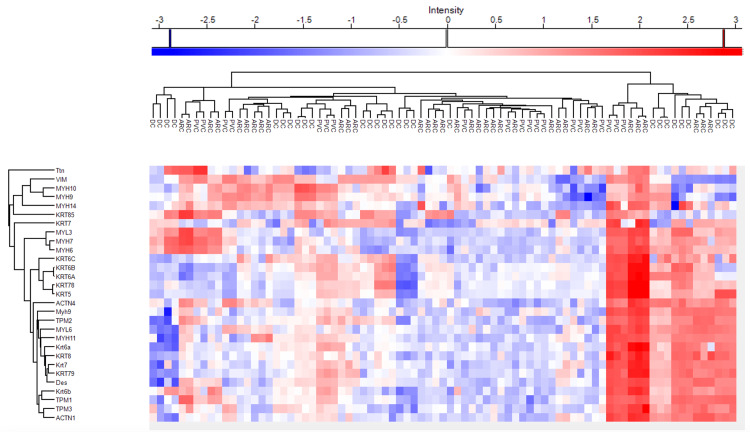
Heatmap representing main cytoskeletal proteins in anterior capsule samples of age-related, diabetic and post-vitrectomy cataract.

**Figure 6:**
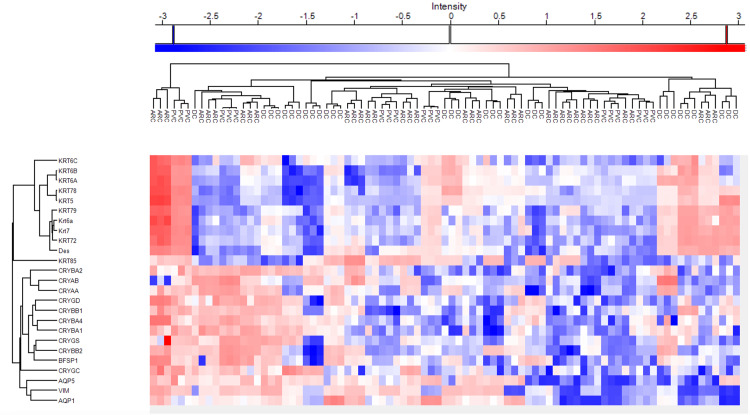
Heatmap representing main cytoskeletal proteins in phaco cassette content samples of age-related, diabetic and post-vitrectomy cataract.

Figures 5-6 demonstrate the heatmaps of the main cytoskeletal proteins in anterior capsule samples and phaco cassette content samples of age-related, diabetic, and post-vitrectomy cataracts.

## Discussion

The preliminary results of our study showed that AQP1, AQP5, BFSP1, BFSP2, CRYAA, CRYAB, CRYBA1/2/4, CRYBB1/2/3, CRYGC, CRYGD, CRYGS, KRTs, and VIM were downregulated in the ARC group. BFSP1 is believed to play a key role in the maintenance of lens transparency, and its downregulation in ARC samples from our study supports this idea [5,7]. CRYAA acts as a chaperone that prevents the aggregation of βγ-crystallins. CRYAA was downregulated in the ARC group, reinforcing its role in ARC formation. Various studies have shown that aquaporins contribute to the maintenance of lens transparency, and the results of our study, with the downregulation of AQP1 and AQP5 from both the anterior capsule and the phaco cassette content samples of the ARC group, are consistent with these data [8].

Protein homeostasis, or proteostasis, is the process that controls protein balance in a cell in order to preserve its healthy state [9]. Lens proteins do not turnover, and lens polypeptides break down over time [9]. The crystallins undergo extensive post-translational modifications (PTMs) with age in order to maintain protein-protein interaction and, thus, their soluble status for lens clarity. These PTMs include truncation, oxidation, deamidation, acetylation, phosphorylation, and glycosylation [5,10]. Crystallin PTMs accumulate with time. If a certain threshold is surpassed and the proteostatic process is unable to maintain proteome balance, this accumulative effect of crystallin PTMs leads to severe imbalances of protein modifications and interactions, thus inappropriate protein association, aggregation, insolubilization, and lens opacification [10]. Cataract formation has been associated with protein aggregation, protein-protein crosslinking, protein insolubilization, and proteome imbalance [10]. Post-translational modifications can be divided into two groups. The first group includes protein modifications caused by cellular metabolites that attach to proteins, with the formation of advanced glycation end products being a great example. The second group includes the spontaneous decomposition of amino acids in proteins as a result of intrinsic amino acid instability, particularly of Ser, Asp, Asn, Ser phosphate, and a lower level of Thr, Thr phosphate, and Cys [9].

Crystallin solubility requires antioxidant conditions. Thus, oxygen levels play a key role in cataract formation. Oxidation-induced modifications of crystallins include acidification, fragmentation, disulfide cross-linking, cleavage of the C-terminal, aggregation, and precipitation [11]. As a result, low oxygen levels in the crystalline lens are necessary to protect its transparency. However, oxygen is present in the lens, but there is a steep gradient from the lens cortex into the lens nucleus [12,13]. Mitochondria consume 90% of this oxygen, and in that way, nuclear O_2_ levels remain low. In mitochondria, oxygen turns into a superoxide anion ($\mathrm{O_{2}^{-}}$) [12,13]. Free radicals are also produced by external factors such as UV light, smoking, and alcohol consumption. These free radicals can actively react and harm lens fiber molecules (DNA, proteins, membrane lipids) [14]. Only some of this damage can be repaired, while the permanent damage from this free radical reaction may cause protein polymerization, cross-linking, and insolubilization [14]. In the lens, there are enzymes (superoxide dismutase, catalase, and glutathione) that protect against superoxide anion, thus preventing free radicals and oxidative damage [15]. Superoxide dismutase catalyzes the destruction of the superoxide anion and produces hydrogen peroxide, which in turn is destroyed by catalase. Glutathione (GSH) is oxidized and forms glutathione disulfide (GSSG), and then it is again transformed to GSH using NADPH [16]. GSH is the most important antioxidant in the crystalline lens, but the antioxidant effectiveness of GHS is lost over a certain threshold of GSH concentration [16]. GSH loss seems to play a key role in cataract formation, and this may be an explanation for the loss of GSH and increased levels of GSSH in cataracts [16]. Vitamin C, vitamin E, and ascorbic acid are also present in the lens and act as antioxidant factors [16].

There is more evidence that protein oxidation seems to be involved in cataract formation and that the degree of protein oxidation is correlated with the grade of cataract, supported by the fact that in the most mature cataracts, a vast majority (>90%) of protein sulfhydryl (protein-SH) groups are lost and almost 50% of all the methionine residues in the nuclear proteins become oxidized to methionine sulfoxide [13]. Due to small protein turnover in the lens, proteins in the nucleus stay stable, but over time, they begin to denature [13]. The denaturation and aggregation of lens crystallins that occur with age may result in protein insolubilization [13]. Urea and dithiothreitol act as agents that solubilize these insoluble proteins, some of which already exist in early age-related nuclear cataracts (ARNCs) [13]. However, several proteins are insoluble, even in those agents, in mature cataracts, and this causes the yellowish coloring in the lens [13]. These insoluble proteins, cross-linked, represent the majority of proteins in the nucleus of mature cataracts [13,16].

In guinea pig models, hyperbaric oxygen results in cataract formation characterized by a loss of GSH, a loss of protein-SH, and protein insolubilization [11]. In the study of Fan et al., lens-specific suppression of GSH synthesis in the LEGSKO mouse, while keeping all other body functions intact, caused biochemical, biological, and cataractous changes mimicking those of human ARNCs and cysteine residues in crystallins that were associated with cataract severity [17]. Wang et al. extended their previous study to detect which non-crystallin disulfides human and LEGSKO mouse cataracts shared [2,18]. Their results revealed that enzymes with oxidized cysteine at critical sites included GAPDH, glutathione synthase, aldehyde dehydrogenase, sorbitol dehydrogenase, and PARK7 [2,18]. Based on their results, extensive oxidation was also present in microfilament and microtubule filament proteins (tubulin and actins), as well as in lens-specific intermediate filament proteins (BFSP1 and BFSP2, vimentin, and cytokeratins) [2,18]. Most of these proteins were positively associated with the degree of cataract severity [2,18].

Fragmentation of proteins by N- or C-terminals to create numerous peptides is usually non-enzymatic and represents one of the most common irreversible PTMs in the lens [19]. Various sites of cleavage of crystallins and other lens proteins have been reported in previous studies [19]. The truncated forms of various crystallins may often act in a different way from the native polypeptide, suggesting an altered conformation, and this affects their aggregation behavior. Limited C-terminal α-crystallin truncation at Ser172 was reported to increase its function as a chaperone, but further truncation may degrade this function [19]. Moreover, truncated bB1 crystallin, trimmed of N-terminal residues, interacts abnormally compared to the native form, and it is suggested that since truncation occurs extensively in the aging human lens, it may be an important factor for age-related cataract formation [19]. In a recent study, it was shown that αB- and βA4- crystallin were upregulated with the loss of lens transparency and that truncation of those specific crystallins increased as the lens opacification advanced [13,20,21]. Other lens proteins that are truncated with age include connexins and cytoskeletal proteins [22]. AQP0 plays a key role in preserving the ordered architecture of the lens and its transparency [22]. The truncated protein AQP0 1-243 maintains water permeability, but further truncation and removal of the C-terminal residues may change the regulation of channel activity, its localization in the fiber cell membrane, or its structure [22]. 

The most abundant PTM in long-lived proteins is racemization, and the major amino acids involved in racemization include Asp, Asn, and Ser, followed by Thr and Phe [9,23]. The main forms of isomerization/racemization of amino acids include the transformation of L- to D-amino acids through the deletion of the hydrogen atom attached to the α-carbon atom and intramolecular cyclization [9,23]. Amino acids, such as Ser, Thr, and Phe, are racemized through the first and simpler mode, while for Asn and Asp, the primary route of racemization is the second and more complex one, and the produced isoforms are L-Asp, D-Asp, and L-isoAsp and D-isoAsp [9]. Researchers investigated the racemization of two residues in A-crystallin, Ser 59 and Ser 62, and found that while D-Ser increased linearly with age in normal lenses, it significantly increased in cataract lenses [24]. These age-related changes are suggestive of the denaturation of α-crystallin and, therefore, its ability to act as a chaperone [21,24]. Several studies investigated Asp racemization. Regarding Asp 151 in αA crystalline, even though substantially racemized in aged human lenses, no significant difference was noted when comparing cataracts and normal lenses [2]. In contrast, in γS-crystallin, the degree of the conversion of Asn 76 to isoAsp in cataract lenses was approximately double compared to normal lenses [2,21,25]. Regarding racemization of Asp 58 in αA-crystallin, levels of d-isoAsp were substantially upregulated in all cataracts compared to age-matched normal lenses [21,26].

Deamidation, which converts an amide to an acid, introducing a negative charge into the protein, is a ubiquitous protein modification and the main PTM for β-crystallins in the aging human lens [27,28]. Accumulated deamidations may cause partial protein denaturation, promote protein aggregation, and disturb inter-protein interconnections [2,27]. In particular, β-crystallins have a compact structure that keeps them stable throughout life, and deamidation, depending on the site, may cause alterations to the stability and solubility of β-crystallins [27]. Deamidation at critical structural regions of β-crystallins, such as the buried interface, causes structural changes significant enough to disturb the stability of the β-crystallins and lead to their aggregation [27,28]. Deamidation enhances the aggregation of soluble β-crystallins, and even though deamidated aggregates are shown not to be large enough to scatter light, deamidations at many sites, occurring with age, may cause further aggregation and induce light scattering [29]. By introducing a negative charge into the hydrophobic sticky region at the interface between domains or subunits, deamidation affects the stability of βB1, βB2, βA3, and γD subunits of the βγ-crystallin family [29].

Moreover, β-crystallins have a compact structure that keeps them stable throughout life, and deamidation, depending on the site, may cause alterations to the stability and solubility of β-crystallins [27,28]. Deamidation at critical structural regions of β-crystallins, such as the buried interface, causes structural changes significant enough to disturb the stability of the β-crystallins and lead to their aggregation [27]. In addition to this, deamidations at the surface affect interactions with other crystallins, possibly due to the added charge and their repulsion [27].

The deamidation of Asn and Gln has been widely studied. Deamidation of Asn and Gln may be involved in crystallin denaturation based on the fact that insoluble proteins that accumulate progressively with age in the human lens have higher levels of deamidation than soluble proteins [9,23,24]. Hains and Truscott characterized the sites of deamidation in all major crystallins in fetal, cataractous, and control human lenses, and they demonstrated that equal numbers of Asn and Gln residues are deamidated in crystallins from aged normal and cataractous lenses [5,30]. Hooi et al. showed that there was no significant difference in the deamidation of Gln92 or Gln170 in γS-crystallin between cataract and age-matched normal lenses [5,26]. Hooi et al. showed that the deamidation of Asp 76 in γS in cataract lenses was approximately double compared to normal lenses, and it seems that a greater extent of deamidation at Asn 76 may be a key feature of human cataracts [2,25].

Almost half of lens αA- and αB-crystallins undergo phosphorylation, and the most common phosphorylation sites are at Ser residues, followed by threonine and tyrosine residues, for both subunits of α-crystallin. The cAMP-dependent pathway phosphorylates the majority of A- and B-crystallins, while non-enzymatic autophosphorylation is a less common pathway [31]. Various studies have investigated the effect of phosphorylation on the chaperone activity of α-crystallin. Phosphorylation of αB-crystallin causes the dissociation of large oligomers into smaller molecules and the reduction of chaperone-like activity [32]. The addition of one or more negative charges to αB-crystallin may lead to an increase in target protein affinity and the rate of subunit exchange, as well as a decrease in crystallin stability and the average oligomeric size [31]. Those alterations to the N-terminal domain region of αA- and αB-crystallin cause structural changes and exposure of the substrate binding sites [31]. Phosphorylation in this region may also interrupt the interactions between the subunits of the crystallin, leading to the dissociation of larger oligomers into smaller oligomers, which are more active [31]. This hyperactivity promotes aggregation, as αA- and αB-crystallin are already saturated and precipitate with the target proteins at an earlier stage [31].

Methylation of Cys in γS-crystallin is particularly important because it blocks disulfide bonding of γS-crystallins, blocks the formation of high-molecular-weight aggregates, inhibits protein insolubilization, and may offer protection against cataracts [21,33]. However, it reaches a plateau early in life and is not affected by cataract development [21,33].

Covalent crosslinking of polypeptides, particularly of γS-crystallin, is another critical PTM. It has been shown that dimeric forms of γS-crystallin and γS-crystallin cross-link to other proteins, including crystallins and cytoskeletal or other membrane-bound structures in older human lenses [34]. Previous studies showed that covalent multimers, which contain a multitude of crystallin fragments, have been found in both the water-soluble and water-insoluble fractions of human lenses, with increasing prevalence in the water-insoluble fraction with age [34]. The covalent interaction between βΒ1- and γS-crystallins might indicate that the two crystallin species are closely located in the lens cytosol, and this might help preserve the solubility of these crystallins [34]. Moreover, there are sites in some residues more susceptible to spontaneous breakdown in long-lived proteins, and these include phosphoserine and phosphothreonine. The protein cross-linking that is mediated by those residues may cause protein aggregation.

## Conclusions

The etiopathogenesis of transparency loss of the crystalline lens can be multifactorial, and age is the major risk factor for cataracts due to the cumulative damage of environmental insults to proteins, and in particular crystallins. Cataract formation involves protein PTMs, protein crosslinking, protein insolubilization, and aggregation, with oxidation being a key. The lens cytoskeleton, microfilament motor activity, microtubule binding, and organizing center seem to be principally involved in cataract formation. It could be stated that cataract is a multifactorial disease due to the role of genetic and environmental factors and its variable clinical presentation, which makes its understanding more complex. Even though multiple studies have investigated this field, more research is required to make prevention or even new non-invasive treatments for age-related cataracts possible.
